# Crystal Structures of the Human G3BP1 NTF2-Like Domain Visualize FxFG Nup Repeat Specificity

**DOI:** 10.1371/journal.pone.0080947

**Published:** 2013-12-04

**Authors:** Tina Vognsen, Ingvar Runár Møller, Ole Kristensen

**Affiliations:** Biostructural Research, Department of Drug Design and Pharmacology, Faculty of Health Sciences, University of Copenhagen, Copenhagen, Denmark; University of Washington, United States of America

## Abstract

Ras GTPase Activating Protein SH3 Domain Binding Protein (G3BP) is a potential anti-cancer drug target implicated in several cellular functions. We have used protein crystallography to solve crystal structures of the human G3BP1 NTF2-like domain both alone and in complex with an FxFG Nup repeat peptide. Despite high structural similarity, the FxFG binding site is located between two alpha helices in the G3BP1 NTF2-like domain and not at the dimer interface as observed for nuclear transport factor 2. ITC studies showed specificity towards the FxFG motif but not FG and GLFG motifs. The unliganded form of the G3BP1 NTF2-like domain was solved in two crystal forms to resolutions of 1.6 and 3.3 Å in space groups P2_1_2_1_2_1_ and P6_3_22 based on two different constructs, residues 1–139 and 11–139, respectively. Crystal packing of the N-terminal residues against a symmetry related molecule in the P2_1_2_1_2_1_ crystal form might indicate a novel ligand binding site that, however, remains to be validated. The crystal structures give insight into the nuclear transportation mechanisms of G3BP and provide a basis for future structure based drug design.

## Introduction

Ras GTPase Activating Protein SH3 Domain Binding Protein (G3BP), a 52 kDa protein highly overexpressed in various cancer tumors [1]–[3], has in the last couple of years proved to be a significant cancer marker protein [4] and a potential drug target [5]–[7]. G3BP is involved in several cellular functions and is implicated for example in stress granule assembly [8], RNA metabolism [9], [10] and hereby cell motility [11], nuclear transportation [12], [13] and NFkappaB [12], Ras [14] and Wnt signaling [15], [16].

There are three human isoforms of G3BP: G3BP1, G3BP2a and G3BP2b [13]. The main difference between the three variants is found in the number of PxxP motifs in the central region of the protein. G3BP1 and G3BP2a have a sequence identity of 65% and contain 466 and 482 amino acids, respectively. G3BP1 and G3BP2 are encoded by genes located on chromosomes 5 and 4 [13], and are, despite their similarities, likely to have different functions. As an example, it has recently been found, that G3BP1 is a negative regulator of wnt/β-catenin signaling [15], whereas G3BP2 is a positive regulator [16].

In addition to the PxxP motifs, G3BP also contains glutamine- and glycine rich regions, an RNA recognition motif (RRM) and an N-terminal NTF2-like domain ranging from residue 11–134 in human G3BP1. The NTF2-like domain is the most highly conserved part of the G3BP sequence and it has been implicated in several G3BP functions. Examples are G3BP dimerisation [8], stress granule assembly [8] and binding to various proliferation-related proteins like Ras GTPase Activating Protein (rasGAP), a negative regulator of ras p21 [13], [14].

The otherwise well-documented binding to rasGAP has recently been questioned based on attempts to reproduce published immuno-precipitation results [17]. Furthermore, another recent publication shows that knockdown of G3BP1 in PCI-15B cells did not affect Ras signalling [18]. This indicates that the suggested rasGAP interaction, if genuine, is complex and dependent on unknown factors.

In addition to rasGAP, the NTF2-like domain contains a binding site for cytoplasmic activation/proliferation associated protein 1 (Caprin-1), a 78 kDa protein tightly associated with stress granules formation and, like rasGAP, cell proliferation [19]. The stable cytoplasmic caprin-1/G3BP complex is likely to be involved in mRNA metabolism [19].

We have previously published the structure of the NTF2-like domain of Rasputin [20], which not surprisingly revealed high structural similarity to nuclear transport factor 2, the namesake of the NTF2-like domain and a small homo-dimer playing an important role in nuclear transport and import of ranGDP to the cell nucleus. The G3BP NTF2-like domain has been suggested to play a similar role in nuclear shuttling. This suggestion is based on findings of G3BP1 and G3BP2 both in the cytoplasm and in the nucleus [1], [2]. Also, mutants of G3BP2a lacking the NTF2-like domain has been shown to be exclusively localized to the cytoplasm [12].

Nuclear transport factor 2 and other NTF2-like domains enter the nucleus via three different repeated nucleoporin motifs: FxFG (where ‘x’ is usually serine), FG and GLFG. Weak binding to these motifs translocates nuclear transport factor 2 and its structurally similar family members through the nuclear pore complex (NPC). Nuclear transport factor 2 has been shown to enter the nucleus via binding to nucleoporins (also called Nups) containing the FxFG motif [21], while the TAP/p15 heterodimer recognizes the FG repeat [22]. Crystal structures of the nuclear transport factor 2 in complex with a peptide containing the FxFG repeat and the TAP/p15 heterodimer in complex containing the FG repeats reveal different binding sites [22], [23]. Despite the different binding sites, recognition of a phenylalanine in a hydrophobic pocket is a common feature.

Here we investigate the specificity of the NTF2-like domain of human G3BP1 to three peptides containing the three nucleoporin motifs FSFG, FG and GLFG, using isothermal titration calorimetry (ITC). Furthermore, we present structures of the NTF2-like domain of human G3BP1. Initially, we obtained hexagonal crystals of G3BP1 10–139 diffracting to around 3.3 Å. While solving and refining the structure, a structure of the G3BP1 NTF2-like domain (PDB ID 3q90) from orthorhombic crystals became available in the Protein Data Bank. We obtained the same orthorhombic crystal form, re-refined the unliganded structure, and solved the structure of a complex of the protein with an FxFG Nup repeat peptide. The resulting structure was subsequently used to improve the lower resolution hexagonal structure.

## Materials and Methods

### Synthetic Peptides

The three synthetic peptides, GQSPGFGQGGSV (referred to as FG), DSGGLFGSK (GLFG) and DSGFSFGSK (FxFG), were obtained as more than 98% pure lyophilized chloride salts from Caslo Laboratory ApS, Denmark.

### Sub-cloning

Sub cloning of the G3BP1 11–139 construct has been described previously [24] and template cDNA coding for human G3BP1 (Swiss-Prot Q13283) was kindly provided by B. Moss [25].

Polymerase chain reaction (PCR) amplification of the DNA was achieved using the following primers: hNTF2.G3BP1-F (5′- CGCGGCAGCATATGGTCGGGCGGGAATTTGTGAGA-3′), and hNTF2.G3BP1-R (5′-CGCGGCAGGCGGCCGCTCAACCAAAGACCTCATCTTGG-3′) for G3BP1 11–139 construct and hNTF2.G3BP1-R and hNTF2.G3BP1-F1 (5′-CATGCTAGCCATATGGTGATGGAAAAACCGAGCCCAC-3′) to generate the G3BP1 1–139.

The PCR products were digested with NotI and NdeI and cloned into the pET28a(+) expression vector (Novagen). All constructs contained a thrombin-cleavable His6 tag, leaving four additional vector-derived N-terminal residues (GSHM) after proteolysis. DNA sequencing (Eurofins MWG Operon) was used for expression vector verification. The expression vector for the G3BP1 1–139 Phe15Ala mutant was produced by GenScript Inc. (USA).

### Protein Production and Crystallization

Purification for all the constructs was carried out as described previously for the Rasputin and G3BP1 11–139 NTF2-like domains [20], [24]. In short, plasmids were transformed into *Escherichia coli* BL21(DE3) cells and protein expressed using ZYM-5052 auto-induction media [26]. The protein was purified by HisTrap HP chromatography, and the affinity-tag was subsequently removed by thrombin cleavage before a final gel filtration (buffer: 10 mM Tris-HCl, pH 8, and 100 mM NaCl). The protein was concentrated by ultrafiltration (Amicon Ultracel 10 K) to 5–10 mg/ml as estimated using a Bradford assay (Bio-Rad).

Hexagonal crystals of the G3BP1 11–139 construct grew within few days in 1.6 M di-ammonium phosphate, 0.1 M MOPS, pH 8, using the hanging drop vapour-diffusion method for crystallization at room temperature. The crystal used for data collection was soaked for three hours in a solution consisting of 2 mM HgCl_2_ and 1.6 M sodium/potassium phosphate buffer, pH 7, in an unsuccessful attempt to obtain a mercury derivative. The crystals were transferred briefly to a solution containing 1.6 M sodium/potassium phosphate, pH 7, and 20% glycerol before flash-cooling in liquid nitrogen. A gold derivative was prepared by addition of KAu(CN)_2_ to the crystallization drop to a final concentration of 2 mM. The crystals were harvested after three days and cryo-protected as described for the native G3BP1 11–139 crystals.

Rectangular crystals of human G3BP1 1–139 were obtained in 20% PEG3350 and 0.1 M Bis-Tris, pH 5.5, using sitting drop vapour-diffusion at 6°C. These crystals grew to a size of 400 µm on the longest edge within a few days. The crystals were cryo-protected by a brief soak in 25% PEG3350, 0.1 M Bis-Tris (pH 5.5) and 10% glycerol before being flash-cooled in liquid nitrogen.

Co-crystallization of G3BP1 1–139 with the FxFG peptide (the molar ratio G3BP/FxFG was 1∶6) under the same conditions as above resulted in long thin needle-like crystals appearing after several weeks from an otherwise clear crystallization drop. These crystals were cryo-protected in the same manner as for native G3BP1 1–139 crystals.

### Structure Determination

X-ray data was collected at beamline I911-2 at MAX-lab (Lund, Sweden). Data processing was carried out using the XDS pipeline [27] in the xia2 software [28]. PHASER [29], as implemented in the CCP4 suite [30], was used for initial structure determination of G3BP1 11–139 in space group P6_3_22 by molecular replacement. The *Cryptosporidium parvum* nuclear transport factor 2 (PDB entry 1zo2, chain A [31]) was used as search model in the absence of a highly identical structure; PDB entry 3q90 became available significantly later. Experimental SIRAS phases obtained from a gold derivative using the program SHARP [32] was used for improvement of electron density maps, but unambiguous tracing of the loops III and VII remained a challenge. Final refinement of the structure was performed using PHENIX [33] software tools (phenix.autosol, phenix.den_refine and phenix.refine). Chain A of the G3BP1 NTF2-like domain in its FxFG peptide binding state (PDB: 4fcm) was used as a reference and for additional restraints in refinement. In addition, SIRAS phases from the gold derivative were used throughout as restraints for the MLHL target function. Automatic B-factor sharpening was applied in calculation of electron density maps before manual rebuilding in the program Coot [34]. The phosphate included in the model occupies a special position and coordinates for its atoms were kept fixed in refinement and alternative orientations of the ion were ignored.

The structures of G3BP1 1–139 with and without the FxFG peptide bound were solved in space group P2_1_2_1_2_1_ by molecular replacement and refined using the PHENIX software suite [35]. An existing 1.7 Å crystal structure of the NTF2-like domain of human G3BP1 (PDB: 3q90) was used as search model. The program Coot [34] was used for manual rebuilding and placement of the FxFG ligand, which was only included in the peptide complex after convergence of refinement. The structure models were validated using the program MolProbity [36] and graphical figures were prepared in PyMOL (The PyMOL Molecular Graphics System, Version 1.3, Schrödinger, LLC).

### Isothermal Titration Calorimetry

ITC measurements for the wild type NTF2-like domain were performed with an ITC200 titration calorimeter (MicroCal). Data analysis was done using the Origin software. Protein samples were dialyzed against 0.1 M NaCl and 0.1 M HEPES, pH 8, and passed through 0.22 µm centrifugal filter units (Millipore) before the experiments. Peptides were solubilized in the dialysis buffer and the pH adjusted before filtration. In each experiment, aliquots of 2 µL peptide (5.2 mM) were injected into the sample cell every 180 seconds at 25°C. Background measurements were performed with buffer injected into the protein solution and peptides injected into the buffer solution. The concentrations of the peptides were determined by amino acid analysis (Department of Systems Biology, DTU, Denmark). Experiments were repeated three times and the results given as mean ± standard deviation.

Data for the Phe15Ala G3BP1 mutant were collected in a single experiment on a VP-ITC (MicroCal) instrument using buffer conditions as for the wildtype. Aliquots of 15 µL peptide (4.1 mM) were injected into the protein sample every 360 seconds at 20°C.

### Accession Numbers

Crystallographic coordinates and structure factors have been deposited at the Protein Data Bank with accession codes 4iia (unliganded form in space group P6_3_22), 4fcj (unliganded form in space group P2_1_2_1_2_1_) and 4fcm (FxFG complex).

## Results and Discussion

### Structure Determination

Two crystal structures of the G3BP1 NTF2-like domain, residues 11–139 and 1–139, were determined to resolutions of 3.3 and 1.6 Å, respectively. The 3.3 Å crystal structure of G3BP1 was solved in space group P6_3_22 with one molecule in the asymmetric unit whereas two molecules describe the 1.6 Å structure in space group P2_1_2_1_2_1_.

The low resolution structure was determined using combined molecular replacement and SIRAS phasing. The structure was refined to final R_work_ and R_free_ values of 30 and 36%, respectively, including 131 protein residues and one phosphate ion. Two loop regions, Gly43-Ala52 (loop III) and Pro116-Phe124 (loop VII), proved difficult to model but the electron density, including an un-biased density modified SIRAS map, suggested that these loops could tentatively be modeled, providing at least an approximate location. The phosphate ion occupies a special position in the crystal lattice, surrounded by six symmetry related copies of Arg107.

**Figure 1:**
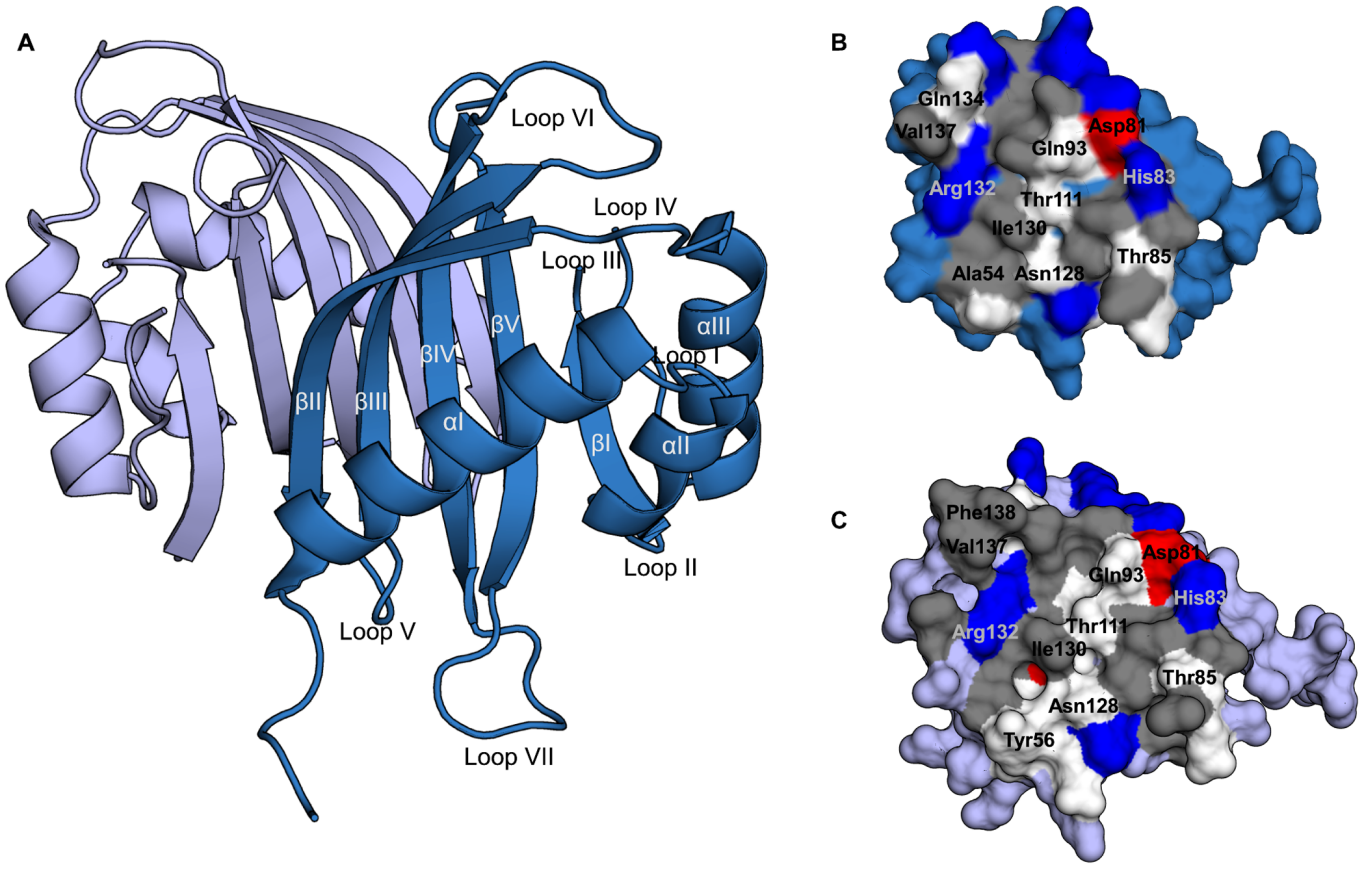
Structural overview. (a) Cartoon representation of the human G3BP1 NTF2-like domain. Chain A: skyblue, chain B: slate (b) Dimer interface, chain A. (c) Dimer interface, chain B (color coding for interface residues; grey: hydrophobic, red: acidic, blue: basic, white: polar).

The structure of G3BP1, residues 1–139, was solved by molecular replacement using an unpublished but deposited structure of the G3BP1 NTF2-like domain (PDB ID 3q90) as search model. Chain A and B could be traced from residue 1 to 137 and 1–138, respectively, with exception of the loop residues 43–51 (loop III in Figure 1a) in chain A and residues 46–50 (loop III) and 117–123 (loop VII) in chain B. Notably, our structure determination models the N-terminal residues 1–7 of both chain A and B, while the N-terminal part was only visible for chain B in the PDB deposited structure (3q90). In addition to chain A and B, 289 water molecules and one glycerol molecule were included in the model. The structure was refined using seven TLS groups to a final R_free_ value of 23% and structure validation showed good overall stereo-chemistry with no Ramachandran outliers (Table 1). Chain A and chain B are almost identical with rmsd of 0.453. The 1.6 Å resolution structure solved in space group P2_1_2_1_2_1_ is used as a reference in the following discussion unless otherwise noted. Statistics for the data processing and model refinement are summarized in Table 1.

**Table 1:** Data reduction and refinement statistics.

*Data processing*(PDB entry code)	Unliganded(4iia)	unliganded(4fcj)	FxFG complex(4fcm)
Space group	P6_3_22	P2_1_2_1_2_1_	P2_1_2_1_2_1_
a, b, c (Å)	89.40, 89.40, 70.14	42.62, 71.40, 87.99	42.78, 71.49, 89.50
Monomers per asymmetric unit	1	2	2
Resolution range (Å)	29.35-3.30 (3.39-3.30)	30.61-1.62 (1.66-1.62)	28.38-2.69 (2.76-2.69)
Unique reflections	2709 (195)	34385(2447)	7987 (566)
Multiplicity	10.0 (10.1)	6.5 (6.2)	3.9 (4.1)
Data completeness (%)	98.7 (99.1)	98.6 (96.2)	99.3 (99.9)
Rmerge (%)	8.5 (>100)	6.7 (63.1)	12.9 (94.3)
I/σ(I)	15.7 (1.5)	14.5 (2.8)	10.0 (1.8)
*Refinement*			
Rwork/Rfree	30.2/35.9	0.1818/0.2314	0.2252/0.2822
Atoms (non-H protein/solvent/ions)	1060/0/1	2156/233/6	2236/62/5
r.m.s.d. bond length (Å)	0.006	0.008	0.003
r.m.s.d. bond angle (°)	0.551	1.081	0.589
Mean B-value (Å2) (protein/solvent)	182.5	29.8/40.4	48.1/32.1
Ramachandran plot (%) (favored/additional/disallowed)	88.4/9.3/2.3	98.0/2.0/0	95.5/4.1/0.4

Values in parenthesis refer to the highest resolution shell.

### Overall Structure of the NTF2-like Domain and Dimer Formation

The NTF2-like domain forms a homo-dimer with each molecule composed of three alpha helices (αI–III) lined up against a beta sheet made up by five beta strands (βI–V) (Figure 1a). The overall structure is highly similar to published structures of the nuclear transport factor 2 (rmsd 1.3 for PDB ID 1zo2) and the NTF2-like domain of Rasputin (rmsd 0.7 for PDB ID 3ujm) as confirmed by a DALI search [37].

Analysis by PDBePISA [38] reveals, that the dimer interface covers a buried area of 1362 Å^2^ and is formed by four salt bridges and 15 hydrogen bonds in addition to hydrophobic interactions. It has previously been suggested, that hydrophobic interactions are pivotal for dimer formation of NTF2 domains [8]. However, Thr85, Asn128 and Arg132 in G3BP1, which are the only residues with side chains directly involved in dimer formation and conserved between nuclear transport factor 2, Rasputin and G3BP1, are all polar. This indicates that polar contacts are more important for dimer formation than previously anticipated.

The NTF2-like domain is known to be crucial for dimerisation of G3BP [8]. However, phosphorylation of Ser149, positioned 16 residues from the C-terminal end of the NTF2-like domain, has been reported to inhibit dimerisation [8]. Thus, the NTF2-like domain is not the only determining factor in dimerisation. It is possible that the residues following the C-terminal of the NTF2-like domain are also involved in or interfere with dimer formation through interactions with β-strands II and/or III.

### Crystal Packing of N-terminal Residues

**Figure 2:**
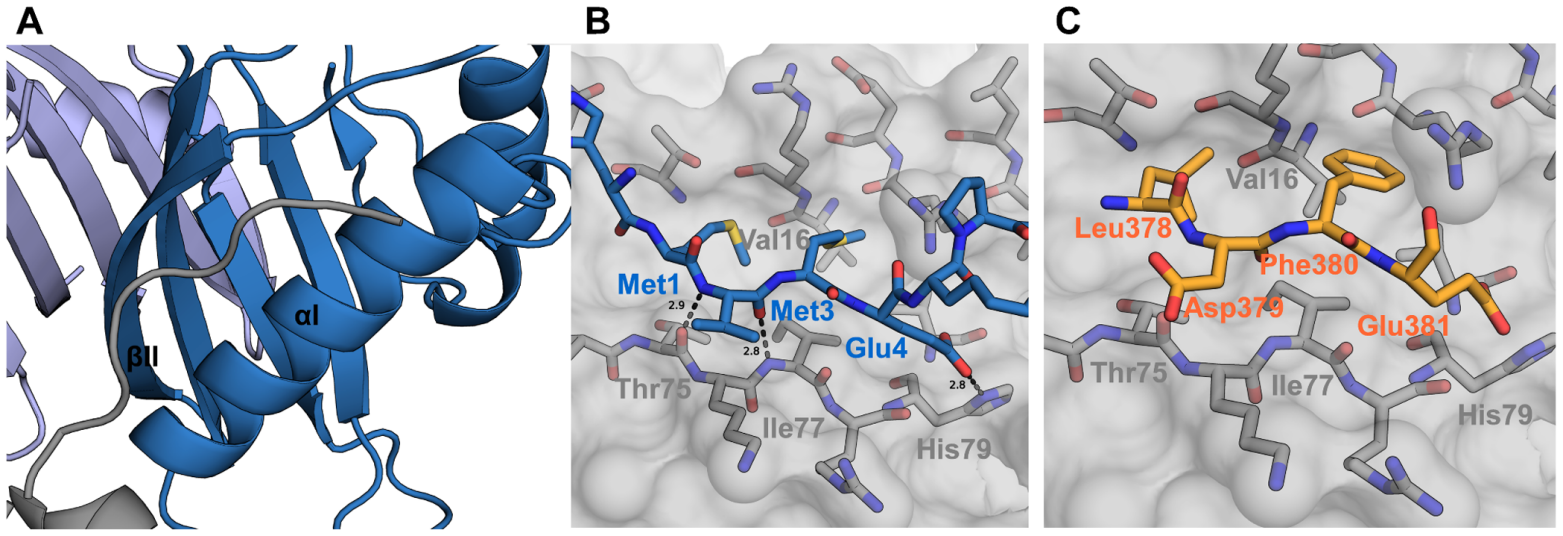
Crystal packing and implications. (a) Cartoon representation of binding of N-terminal residues from a symmetry related molecule (grey) to chain A (skyblue). (b) Close-up of the binding of N-terminal residues. (c) Manual docking of four residues, residues 378–381, from the minimum binding motif of Caprin-1.

Initially, we were focusing our studies on the G3BP1 11–139 construct under the assumption that the N-terminal residues would be disordered and likely to interfere with crystal packing. When a 1.7 Å structure of G3BP1 1–139 was deposited in the Protein Data Bank (PDB ID 3q90) by the Structural Genomics Consortium, this anticipation was revised and the 1–139 construct included in our work. By grid-screening around the crystallization conditions provided for the 3q90 structure, we optimized the crystals and were able to increase the resolution slightly (from 1.7 to 1.6 Å) and furthermore model all N-terminal residues from both chains. The difference in resolution and space group between G3BP1 1–139 and 11–139 can be assigned to crystal packing effects largely caused by the N-terminal residues. In the G3BP1 1–139 structure, residues 1–4 are accommodated in a hydrophobic ridge between αI and βII of a symmetry related molecule as shown in Figure 2. The N-terminal residues are held in place by burying methionine residues 1 and 3 in the hydrophobic ridge. Furthermore, a salt bridge exists between Glu4 and His79. Crystal packing is denser in the G3BP1 1–139 structure as compared to the 11–139 structure with estimated solvent contents of 42 and 56 percent, respectively. Despite being a probable crystal artifact, the binding of the N-terminal residues to a symmetry related molecule could indicate a possible ligand binding site. This could be validated by a ligand site prediction search using the LIGSITE^CSC^ server [39], where the ridge between αI and βII was predicted as one out of two potential ligand binding sites. The fact that we in our study observe this feature for both molecules in the asymmetric unit provides support to its significance as a tentative mimic of an additional and new protein binding site of the G3BP1 NTF2-like domain.

Although it remains to be experimentally validated, we speculate that the G3BP1 interaction partner, Caprin-1, might be recognized at this site. A 10 residue long peptide (FIQDSMLDFE) has been identified as the minimum motif for binding of Caprin-1 to G3BP [19]. Manual docking of the latter four residues from this peptide into the electron density of residue 1–4 of the G3BP1 structure as shown in Figure 2c retains both the hydrophobic interactions and the salt bridge to His79. Other interaction partners, like USP10 and Tudor-SN [40], [41], might also be recognized by this site but information about their binding motifs is limited.

### G3BP1 Binds the FxFG Peptide but not FG and GLFG Peptides

**Figure 3:**
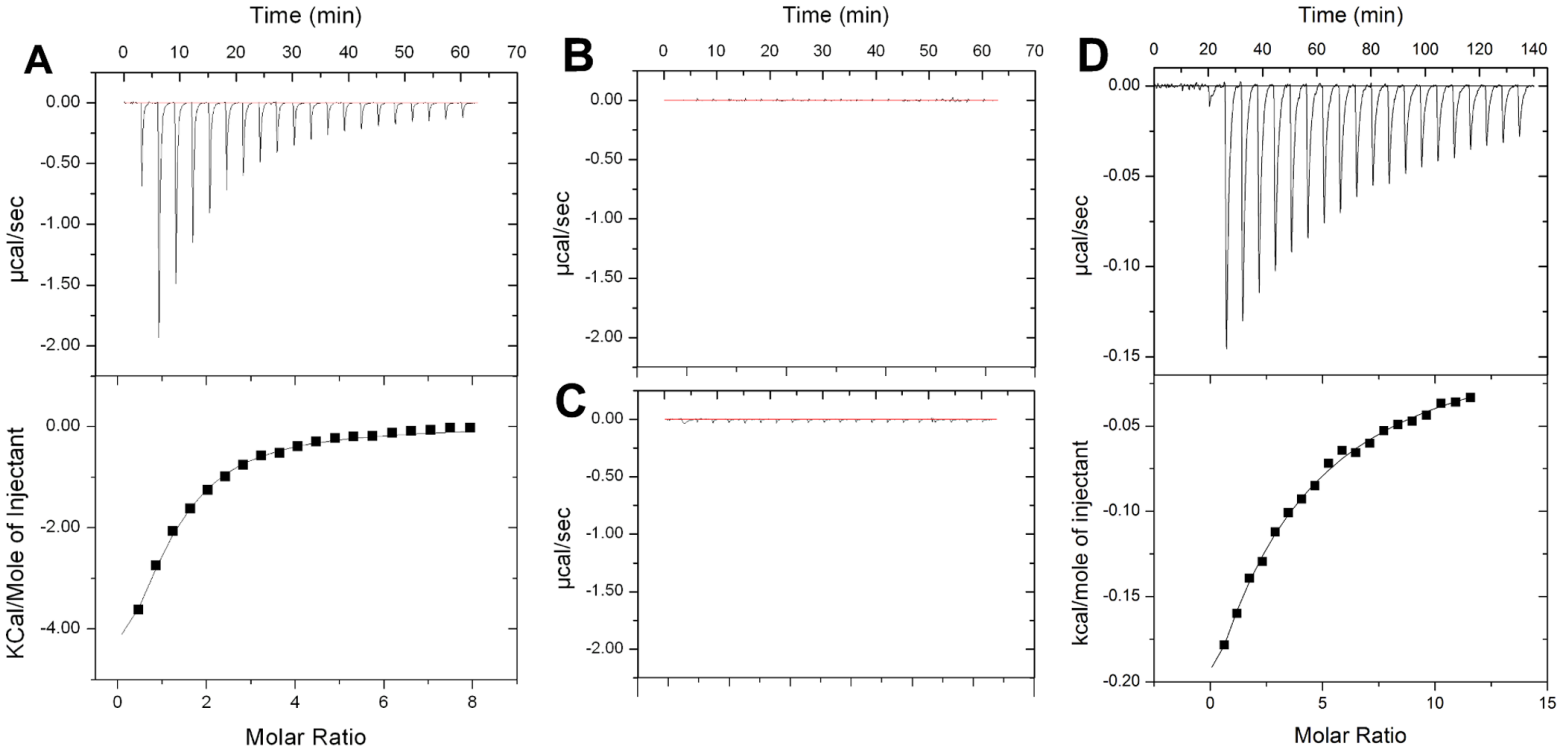
Isothermal calorimetric titrations for binding of Nup peptides to the G3BP1 NTF2-like domain. (a) Titration of the FxFG peptide to G3BP1 1–139 (upper panel: raw data, lower panel integrated heat of binding). (b) Titration of FG peptide into G3BP1 1–139 (raw data). (c) Titration of GLFG peptide into G3BP1 1–139 (raw data). (d) Titration of FxFG peptide to the G3BP1 1–139 Phe15Ala mutant (upper panel: raw data, lower panel: integrated heat of binding).

Binding of nucleoporin peptides to the G3BP1 NTF2-like domain was studied by isothermal titration calorimetry, using sequences representing three known motifs:, FG (GQSPGFGQGGSV), GLFG (DSGGLFGSK) and FxFG (DSGFSFGSK). The same peptides have been used in structural studies of other NTF2 domains [22], [23], [42]. While titration of the FG and GLFG peptides into both G3BP1 1–139 and G3BP2a 1–139 (data not shown) did not result in significant heat changes, titration of the FxFG peptide resulted in relatively large exothermic heat changes and exhibited typical binding characteristics (Figure 3).

The heat changes was fitted to a one-binding-site per chain model based on the crystal structure described below, and a K_d_ value of 115±3 µM was calculated for the G3BP1 NTF2-like domain. A similar binding affinity was found for the G3BP2a NTF2-like domain (K_d_ = 114±2 µM). For comparison, a dissociation constant in the range 20–50 µM has been reported for binding of a single FxFG motif to nuclear transport factor 2 [43], [44]. Hence, G3BP appears to have a lower affinity than nuclear transport factor 2 for this nucleoporin repeat motif but it is still clearly selective.

### The FxFG Motif is Recognized in a Hydrophobic Pocket between αI and αII

**Figure 4:**
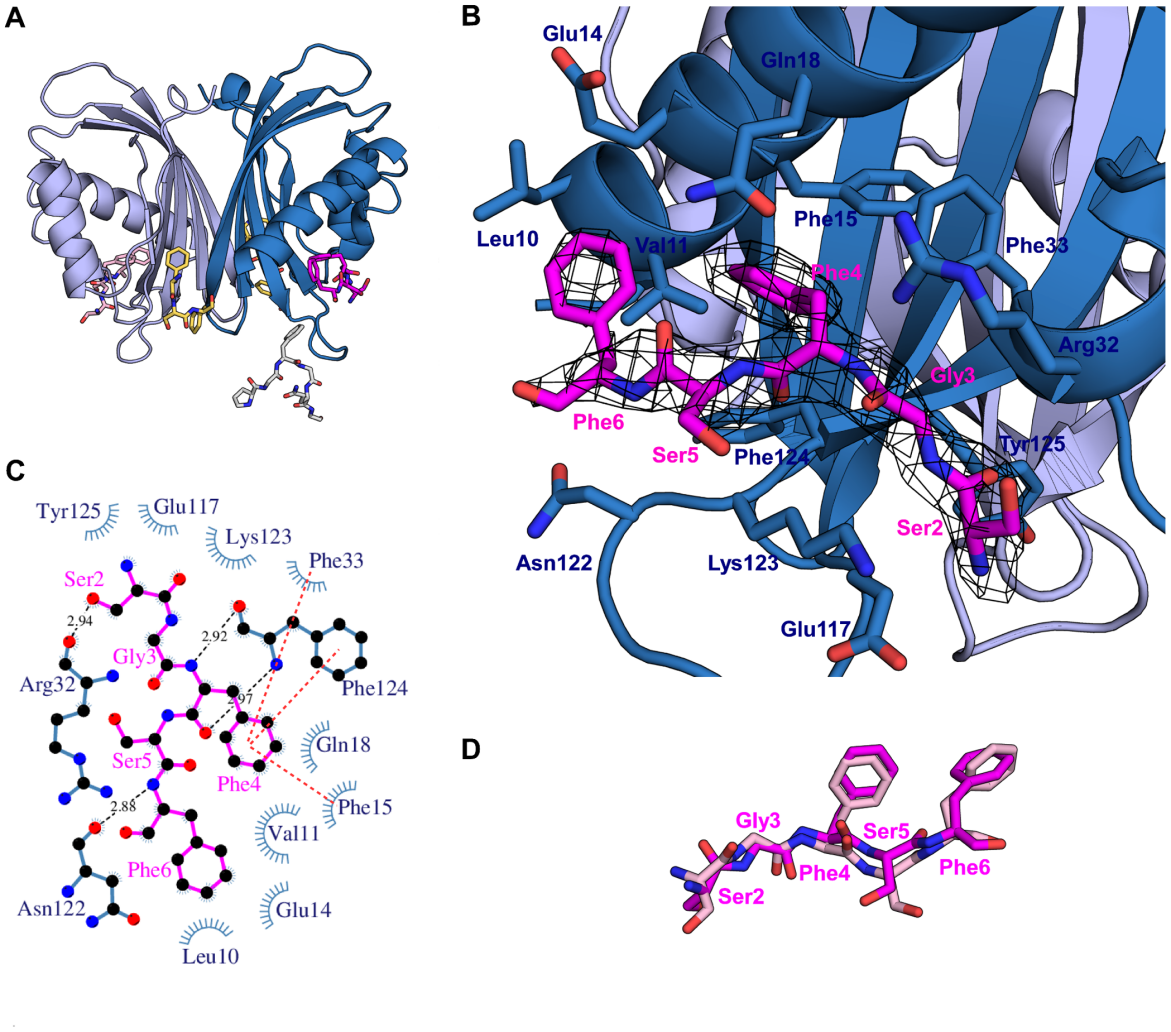
Nuclear transport. (a) Cartoon representation of the peptide complex. The FxFG peptide is shown as pink sticks. The positions of an FxFG peptide in yeast nuclear transport factor 2 (yellow sticks, PDB ID 1gyb) and an FG peptide in the TAP-p15 complex (gray sticks, PDB ID 1jn5) are indicated. (b) Close up of the peptide binding site. 2Fo-Fc electron density map contoured at sigma 1 is shown for the FxFG peptide and hydrogen binding and salt bridge distances are given in Ångstrom. (c) Schematic LIGPLOT overview of interactions between G3BP1 (blue) and the FxFG peptide (pink). Hydrogen bonds are indicated by black dashed lines, hydrophobic contacts are represented by arcs with spokes radiating towards the peptide and pi-stacking is indicated by red dashed lines. (d) Alignment of the FxFG peptides from chain A (pink) and chain B (light pink).

Co-crystallization of G3BP1 1–139 with the FxFG peptide under the same conditions as for G3BP1 1–139 alone resulted in long needle-like crystals that differed significantly in morphology from the rectangular crystals obtained for the unliganded form of G3BP1 1–139. The crystals diffracted to 2.7 Å and the structure was solved by molecular replacement using the unliganded structure (PDB ID 4fcj) as search model. Electron density corresponding to the FxFG peptide was unambiguously located in a hydrophobic pocket between helices αI and αII in chain A and chain B, and residues 2–5 (SGFSF) of the peptide was subsequently modeled and included in refinement (Figure 4a–b). All residues, 1–139, were modeled in chain A, while residues 47–49 remain disordered in chain B. Thus, more residues could be modeled in the peptide complex than in the unliganded structure. This suggests that the NTF2-like domain and in particular loops VI and VII become more ordered upon binding of the peptide. Apart from this, no major changes in backbone and side chain conformations are observed, and an all-atom superposition of the unliganded structure and the ligand bound form provides a calculated rmsd of 0.44 Å. The model was refined using seven TLS groups to an R_free_ of 28%. Removal of the peptide chains from the structure results in an increase of the R_free_ value to 29.5%.

The peptide adapts a β-strand conformation, where the N-terminal phenylalanine, Phe4, from the FxFG motif is held in place by G3BP through parallel-displaced pi-stacking with Phe15 and Phe124, and T-shaped pi-packing with Phe33 from G3BP1. The second phenylalanine, Phe6, in the peptide forms hydrophobic contacts to Leu10, Val11 and the Cβ and Cγ atoms of Glu14. In addition to the hydrophobic interactions, four hydrogen bonds from the backbone of the peptide to G3BP1 exist. Due to the almost palindromic character of the peptide sequence (DSGFSFGSK), these hydrogen bonds were decisive in determining the correct orientation of the peptide during modeling. The two serine residues of the peptide are exposed to the solvent. Interestingly, the glycine residue contained in the FxFG repeat could not be modeled into electron density, suggesting that it may mainly be important to provide structural flexibility and not as a contributor of binding energy.

The orientation of the FxFG peptides is very similar between the two chains as shown in Figure 4c. Especially, the orientation of Phe4 is highly conserved between peptide chains C and D, indicating that this residue is highly important. Both the FG and GLFG peptides contain a phenylalanine residue at this position, but the latter does not bind the NTF2-like domain of G3BP1. The lack of GLFG binding is caused by the leucine residue, which cannot be accommodated in the binding pocket between Phe33, Tyr125 of G3BP1 and Phe4 of the peptide. Considering the fact that the FG peptide does also not bind G3BP1, it is reasonable to assume that Phe6 is in addition crucial for binding. The FG peptide contains glutamine at the position equivalent to Phe6 in FxFG. A hydrophobic residue at this position could possibly allow binding. Inspection of the sequences of 10 human Nups (NUP107, NUP133, NUP153, NUP43, NUP37, NUP88, NUP85, NUP53, NUP160 and NUP155) reveals that hydrophobic residues are present at this position in six of ten Nups. Thus, the possibility that G3BP may bind a FG motif cannot be eliminated in cases where the motif is followed by a hydrophobic residue.

In order to exclude the possibility of the binding site being a crystal artifact, Phe15 in G3BP1 was mutated to alanine and the binding affinity for the FxFG peptide measured by ITC. The Phe15Ala mutant showed weaker binding to the peptide with a K_d_ value of 670 µM indicating that the binding site identified in the crystal structure is genuine.

Most of the residues of the NTF2-like domain involved in binding of the FxFG peptide are conserved between G3BPs from various species. An exception is Phe124, which in Rasputin, the *Drosophila melanogaster* variant of G3BP, is a tyrosine residue. Inspection of the crystal structure of the Rasputin NTF2-like domain does, however, reveal that the orientation of Tyr120 in Rasputin is identical to the orientation of Phe124 in G3BP1, and the ability to interact with nucleoporin repeats through pi-stacking is retained at this position. It is therefore likely that binding of FxFG motifs is a common feature of G3BP, which is kept across species and isoforms.

### Nuclear Shuttling of G3BP

**Figure 5:**
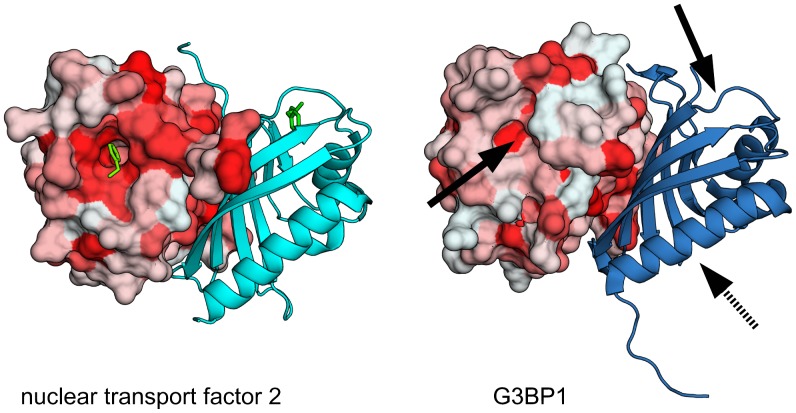
Ran binding. Surface representation of nuclear transport factor 2 (left hand side, PDB ID 1a2k) and the G3BP1 NTF2-like domain (right hand side, PDB ID 4fcj) colored by Eisenberg`s hydrophobicity scale [50]. Phe72 of RanGDP is shown as sticks (green). The corresponding proposed binding sites for Ran at G3BP are indicated by solid arrows. The binding site for the FxFG peptide is indicated by a dashed arrow.

The nucleoporin repeat binding site in G3BP1 differs from that of nuclear transport factor 2 (PDB ID 1gyb) and TAP-p15 (PDB ID 1jn5). Nuclear transport factor 2 binds the FxFG peptide at the edge of the dimer interface, whereas the FG peptide is located at an apical position of the TAP NTF2-like domain close to loop V and VII (Figure 5a). The cellular location of G3BP is phosphorylation-dependent i.e. G3BP1 enters the nucleus as Ser149 becomes phosphorylated [45]. Phosphorylated G3BP is suggested to be monomeric [8], implying that the protein passes the nuclear pore complex as a monomer. This provides a reasonable rationale as to why the FxFG binding site is not located at the dimer interface as in nuclear transport factor 2.

The binding site for the FxFG peptide is coincident with the position of a HEPES molecule in the structure of the NTF2-like domain of Rasputin [20], the drosophila variant of G3BP. This tentatively suggests that Rasputin enters the cell nucleus by a mechanism similar to that of G3BP.

We and others have previously suggested, that binding of RanGDP to nuclear transport factor 2 is retained in G3BP, however with a lower binding affinity [20], [46]. Upon binding to nuclear transport factor 2, Phe72 of Ran is buried in a hydrophobic pocket located at the “top” of the NTF2-like domain [47]. G3BP1 contains a similar hydrophobic pocket that, despite being less hydrophobic and narrower (Figure 5), appears to accommodate Ran binding. In contrast to nuclear transport factor 2, which only binds Ran in its GDP bound state, G3BP has been reported to bind to both RanGDP and RanGTP [46].

G3BP has several reported functions in the nucleus and associates e.g. with the classical marker of active transcription, acetylated histone H3 [1], and it is suggested that G3BP facilitates mRNA export [48]. Furthermore, both G3BP1 and G3BP2 are negative regulators of the tumor suppressing p53 protein and they are able to relocalize p53 from the nucleus to the cytoplasm [49]. Blocking G3BP from nuclear shuttling could, thus, hypothetically regulate gene transcription and be of interest in drug development.

Altogether, the crystal structures and ITC measurements presented here provide insight to NPC mediated transport of G3BP and might support initiatives towards structure based drug design.
